# Post-transcriptional and Post-translational Modifications of Primary Cilia: How to Fine Tune Your Neuronal Antenna

**DOI:** 10.3389/fncel.2022.809917

**Published:** 2022-02-28

**Authors:** Cecilia Rocha, Panagiotis Prinos

**Affiliations:** ^1^Early Drug Discovery Unit, Montreal Neurological Institute-Hospital, McGill University, Montreal, QC, Canada; ^2^Structural Genomics Consortium, University of Toronto, Toronto, ON, Canada

**Keywords:** primary cilia, neurons, epigenetics, RNA splicing, brain tumors

## Abstract

Primary cilia direct cellular signaling events during brain development and neuronal differentiation. The primary cilium is a dynamic organelle formed in a multistep process termed ciliogenesis that is tightly coordinated with the cell cycle. Genetic alterations, such as ciliary gene mutations, and epigenetic alterations, such as post-translational modifications and RNA processing of cilia related factors, give rise to human neuronal disorders and brain tumors such as glioblastoma and medulloblastoma. This review discusses the important role of genetics/epigenetics, as well as RNA processing and post-translational modifications in primary cilia function during brain development and cancer formation. We summarize mouse and human studies of ciliogenesis and primary cilia activity in the brain, and detail how cilia maintain neuronal progenitor populations and coordinate neuronal differentiation during development, as well as how cilia control different signaling pathways such as WNT, Sonic Hedgehog (SHH) and PDGF that are critical for neurogenesis. Moreover, we describe how post-translational modifications alter cilia formation and activity during development and carcinogenesis, and the impact of missplicing of ciliary genes leading to ciliopathies and cell cycle alterations. Finally, cilia genetic and epigenetic studies bring to light cellular and molecular mechanisms that underlie neurodevelopmental disorders and brain tumors.

## Introduction

The primary cilium is a ubiquitous cellular organelle that resembles an antenna whose main function is to sense extracellular signals through a wide repertoire of receptors present on its surface and linked signaling pathways. Once overlooked as an evolutionary vestige, the cilium has recently emerged as the sensory antenna of many pivotal signaling pathways, such as Sonic Hedgehog (SHH), G-protein coupled receptor (GPCR), platelet-derived growth factor receptor (PDGFR), WNT and TGF-β signaling (Akella et al., 2010; Goetz and Anderson, 2010; Nishimura et al., 2019). Primary cilia play a major role in cellular signaling and are required for SHH, PDGF and WNT signaling cascades that are crucial during neuronal development and patterning. Furthermore, stem cells and neural progenitor cells also possess cilia where they play a critical role in cell cycle, proliferation, and differentiation. Ciliary defects lead to different pathologies collectively termed as ciliopathies. In contrast, many tumor cells lose their primary cilia, resulting in an increase in cell proliferation and malignancy. We will begin presenting some concepts in ciliogenesis and discussing the role of primary cilia in brain development and disease before addressing how post-transcriptional and post-translational modification control cilia status contribute to these processes.

## Ciliogenesis

Cilia are microtubule-based structures that emanate from the cell membrane. Whereas motile cilia are found in specialized tissues such as in the respiratory tract and brain ventricles, one primary cilium can be present in every cell. The process to generate a cilium, ciliogenesis, involves several centrosomal proteins and it is tightly coordinated with the cell cycle (Li and Li, 2021). Primary cilia formation can start at G0/G1 phases of the cell cycle, whereas cilia disassembly occurs prior to cell cycle re-entry (Kim and Tsiokas, 2011), and the cell cycle regulation of the primary cilium has been the subject of several detailed reviews (Plotnikova et al., 2009; Kim and Tsiokas, 2011; Goto et al., 2013; Kasahara and Inagaki, 2021; Li and Li, 2021).

Centrosomes are microtubule organizing centers formed by centrioles that are cylindrical structures formed by microtubules that serve as a platform for microtubule polymerization. During mitosis the centrosomes are duplicated and migrate to opposite poles of the cell. After cell division the mother centriole, formed in the previous mitosis, can be dislocated to anchor to the ciliary membrane through the distal appendages. This is an important step to initiate ciliogenesis and, at this point the modified centriole is called a basal body (Kobayashi and Dynlacht, 2011). The basal body is a barrel-like structure where the primary cilia axoneme grows from, with the participation of a specialized transport called intraflagellar transport (IFT) (Ishikawa and Marshall, 2011).

During ciliogenesis ciliary proteins produced in the Golgi can be delivered to the base of the cilium by vesicles as they carry a ciliary target sequence that is recognized by a group of proteins such as Arf4 that regulates the vesicle traffic toward the basal body and the cell membrane. There are other proteins that also participate in this trafficking of vesicles to the cilium such as Rab11, Rab8 and a protein complex called Bbsome that controls IFT assembly and cilia-mediated Hedgehog signaling pathway (Wei et al., 2012; Eguether et al., 2014).

Once vesicles reach the basal body and the cellular membrane, they can be transported along the cilium by IFT proteins that serve as adaptor proteins connecting vesicles to motors that navigate along the axoneme. This process then creates the primary cilium that is a very small and dynamic microdomain in the cell. We will present how primary cilia are involved in different signaling events controlling several processes within the cell such as cell–cell communication, differentiation and cell division (Pugacheva et al., 2007; Gerdes et al., 2009; Goetz and Anderson, 2010; Ishikawa and Marshall, 2011; Kim et al., 2011).

## Cilia in Brain Development

Neuronal development is a complex process requiring neural stem cell activity, lineage commitment and neuronal maturation. These events are controlled by signaling pathways that have been shown to be mediated by the primary cilium and ciliary defects are observed in several human genetic syndromes that are characterized by neurological symptoms highlighting the importance of cilia in neuronal development (Table 1).

**TABLE 1:** Cilia related genes and epigenetic alterations that have been studied in neurodevelopment and cancer.

	Affected gene	Function	Phenotype	Involved tissue or cells	References
**MT PTMs and disease**					
	AGTPBP1 (CCP1)	Deglutamylating enzymes	Childhood-onset neurodegeneration with muscular hypotonia, global developmental delays, and cerebellar atrophy	Cerebellum, spinal motor neurons, and peripheral nerves	Magiera et al., 2018
	AGBL5 (CCP5)	Deglutamylating enzymes	Spermatogenesis disruption and male infertility	Spermatids	Giordano et al., 2019
	CCP1	Deglutamylating enzyme	Upregulated polyglutamylation causes neurodegeneration	Purkinje cells	Rogowski et al., 2010
	Ccp1 and Ccp6	Deglutamylating enzymes	Excessive polyglutamylation causes neurodegeneration	Cerebral cortex	Shashi et al., 2018
	HDAC6	Deacetylase	Targeting HDAC6 could be a suitable strategy to ameliorate cognitive decline observed in Alzheimer’s disease	Brain	Govindarajan et al., 2013
	TTLL3	Tubulin glycylation	Loss of glycylation results in shortening of primary cilia and retinal degeneration	Photoreceptors	Bosch Grau et al., 2017
	TTLL3	Tubulin glycylation	Loss of glycylation results in loss of primary cilia and cancer formation	Colon	Rocha et al., 2014
	TTLL4	Tubulin glutamylation	TTLL4 overexpression in breast cancer cells is associated with brain metastasis	Breast and Brain	Arnold et al., 2020
**Primary cilia and neurodevelopmental disorders**					
	Arl13b	Ciliogenesis	Axonal tract malformation, Joubert Syndrome Related disorder	Projecting neurons	Guo et al., 2019
	BBS1-19	Bbsome	BBS, Joubert Syndrome, Meckel-Gruber syndrome, McKusick-Kaufman syndrome, Senior-Loken syndrome	Multisystemic	Novas et al., 2015
	CEP290	Centrosome	Joubert Syndrome, Meckel-Gruber syndrome, BBS, nephronophthisis, Senior-Loken syndrome	Multisystemic	Coppieters et al., 2010
	CPAP	Centrosome	Seckel Syndrome correlated with microcephaly	Neuronal progenitor	Gabriel et al., 2016
	NEK1	Cilia assembly	Oral-facial-digital syndrome type II, Mohr Syndrome	Multisystemic	Monroe et al., 2016
	OFD1	Ciliogenesis	Oral-facial-digital syndrome type I, CNS abnormalities and cystic kidney disease	Multisystemic	Ferrante et al., 2006
	Smo	HH Signaling	Medulloblastoma	Cerebellum	Han et al., 2009
**Alternative splicing of genes encoding cilia proteins in brain tumors**					
	CDK20 aka CCRK	Cell cycle, ciliogenesis, development	Various cancers (GBM, medulloblastoma, colorectal, ovarian, hepatocellular, lung, ovarian etc.), developmental defects	Brain tumors, epithelial tumors, embryogenesis, neural patterning, eye, lung, skeletal morphogenesis	Snouffer et al., 2017.
	ULK3	Ciliogenesis, autophagy	Bartter syndrome, autophagy, positive and negative regulator of Shh signaling	Brain, respiratory epithelia	Maloverjan et al., 2010
	STK36 aka Fused	Primary cilia, Hedgehog pathway	Hydrocephalus, primary cilia diskynesia	Ubiquitous, gastric tumors, testis, ovaries	Wilson et al., 2009; Han et al., 2019
	NEK1	Cilia assembly	Polycystic Kidney Disease, Oral-facial-digital syndrome type II, Mohr Syndrome, ALS	Kidney, CNS, skeletal, multisystemic	Monroe et al., 2016
**Splicing factors essential for ciliogenesis**					
	PRPF8	Spliceosome snRNP formation	Retinitis Pigmentosa	Retina	Tanackovic et al., 2011b; Wheway et al., 2015
	PRPF6	Spliceosome snRNP formation	Retinitis Pigmentosa	Retina	Tanackovic et al., 2011a;
	PRPR31	Spliceosome snRNP formation	Retinitis Pigmentosa	Retina	Buskin et al., 2018
	SON	Splicing factor	ZTTK multiple congenital anomalies-mental retardation syndrome	Brain	Stemm-Wolf et al., 2021
	SANS/USH1g	Ciliary scaffold protein	Usher Syndrome	Inner ear, cochlear hair cell bundles	Yildirim et al., 2021
	SRSF1	Splicing factor	Cancer, autoimmune disease	Ubiquitous	Haward et al., 2021
	MAGOH	Exon junction complex core factor	Microcephaly neural stem cells	Neural stem cells	Kwon et al., 2021
	RBM8A	Exon junction complex core factor	Thrombocytopenia-Absent radius (TAR) syndrome, microcephaly	Limb development, hematopoiesis, brain development	Kwon et al., 2021
	EIF4A3	Exon junction complex core factor	Robin sequence, ccenter mandible, and limb anomalies (Richieri-Costa-Pereira syndrome), microcephaly	Craniofacial, limb and brain development	Kwon et al., 2021
**Epigenetic factors involved in ciliogenesis**					
	HDAC6	Epigenetics, histone and tubulin deacetylation	Chondrodysplasia, brachydactyly, hydrocephaly	ubiquitous	Hubbert et al., 2002; Pugacheva et al., 2007; Smith et al., 2018
	HDAC3	epigenetics, histone deacetylation	Circadian rythms, metabolism, cardiomyopathy	Ubiquitous	Park et al., 2019
	HDAC8	Epigenetics, histone deacetylation	Cornelia de Lange mental retardation syndrome	Brain	Park et al., 2019
	HDAC2	Epigenetics, histone deacetylation	Cancer	Ubiquitous	Kobayashi et al., 2017
	SIRT2	Histone and protein deacetylase	Anoxia, Wallerian degeneration, aging	Ubiquitous	Zhou et al., 2014
	ATAT1	*a*-tubulin acetyltransferase	Forebrain development, motor neurons	Brain	Akella et al., 2010; Shida et al., 2010
	WDR5	Histone modifications, MLL complex	Kcenterrsa syndrome, Kabuki syndrome, center-right asymetry	Ubiquitous	Kulkarni and Khokha, 2018
	EZH2	H3K27 histone methyltransferase	Weaver syndrome, mantle cell lymphoma, leukemia, breast cancer, melanoma, prostate cancer	Ubiquitous	Zingg et al., 2018
	SMYD2	Protein methyltransferase	Cancer, cardiovascular	Ubiquitous	Li et al., 2020
	MLL2	H3K4 methyltransferase	Leukemia, dystonia 28-childhood onset	Ubiquitous	Yang et al., 2019
	G9A/GLP	H3K9 methyltransferase	Embryonic development, cognition-adaptive behavior, obesity	Ubiquitous	Shin et al., 2015

Primary cilia participate in neuronal development as they are crucial for early brain patterning, neurogenesis, neuronal maturation, and survival mostly through regulating cell cycle progression and key developmental signaling pathways such as SHH and WNT (Guemez-Gamboa et al., 2014; Youn and Han, 2018). Moreover, severe brain alterations are found in different diseases related to primary cilia disfunction (Table 1). Importantly, alterations and disruption in ciliogenesis dysregulate signaling events that are implicated not only in several neurodevelopmental diseases but also brain tumors, highlighting the importance of cilia in brain development (Table 1).

The SHH signaling pathway guides brain development and SHH signals depend on proper primary cilia IFT machinery to be carried in and out of the cilium. The SHH pathway regulates cell fate and self-renewal, and it is activated in the cilium. In the presence of SHH ligand, the receptor Smoothened is recruited to the cilium promoting the formation of activated forms of GLI (GLIA) that migrate into the nucleus and modulate target genes (Anvarian et al., 2019). It was demonstrated that disruption of SHH signals in IFT mouse mutants lead to neural tube and patterning defects (Huangfu et al., 2003) such as disorganized midline (Gazea et al., 2016).

Besides the SHH signaling pathway that is modulated by the primary cilium, WNT signaling also plays an important role in brain development. The WNT pathway controls cell proliferation, differentiation, migration, apoptosis and tissue morphogenesis (Ng et al., 2019). The involvement of cilia in WNT signaling is not as well studied as SHH but some studies have shown that the protein inversin plays a crucial role in the planar cell polarity (PCP) pathway by recruiting disheveled to the plasma membrane upon WNT receptor Frizzled activation (Simons et al., 2005; Lienkamp et al., 2010). The primary cilium was also shown to act as a regulator of canonical WNT signaling in kif3a knockout embryos that lack cilia and display overactivation of WNT reporter (Corbit et al., 2008).

## Cilia, Neural Stem Cells, and Neural Progenitors

Neuronal formation requires proliferation of neuronal stem cells, cellular migration, and differentiation. There are different models of cell division observed in neuronal progenitors such as self-amplifying division to produce two progenitors, self-renewing division to produce one progenitor and one differentiating cell, and self-consuming division to produce two differentiating cells. Importantly, cell proliferation and the exit of the cell cycle are crucial for neurogenesis as it determines the size of the brain and the formation of neuronal and glial cells (Ohnuma and Harris, 2003).

Primary cilia assemble and disassemble during the different phases of the cell cycle functioning as a barrier to cell cycle progression, therefore cilia defects affect proliferation and differentiation of neural progenitors leading to developmental malformations and diseases (Youn and Han, 2018), notably microcephaly and learning disabilities as summarized in Table 1.

Mutations in ciliary proteins are associated with ciliary disruption and cell cycle alterations. Disruption in WDR62 protein, cause of microcephaly in humans (Bilguvar et al., 2010; Bhat et al., 2011; Kousar et al., 2011; Zombor et al., 2019) regulates centriole’s activity and cilia disassembly. WDR62 localizes to the basal body (Zhang et al., 2019) and is involved in IFT88 recruitment to the cilium (Shohayeb et al., 2020) indicating its involvement in ciliogenesis. The importance of WDR62 protein in brain development was studied in murine and human cerebral organoids. *WDR62*-deficient organoids displayed smaller brain sizes as a result of the depletion of neuronal progenitors (Zhang et al., 2019). Microcephaly was associated with defective NPC population that presented impaired cilia disassembly leading to increased cilia length and defective proliferation and differentiation. Defective cilia length and progenitor proliferation was rescued by *Kif2a* overexpression highlighting the importance of the ciliary proteins WDR62 and KIF2A in cilia disassembly and NPC maintenance during brain formation (Zhang et al., 2019).

**FIGURE 1:**
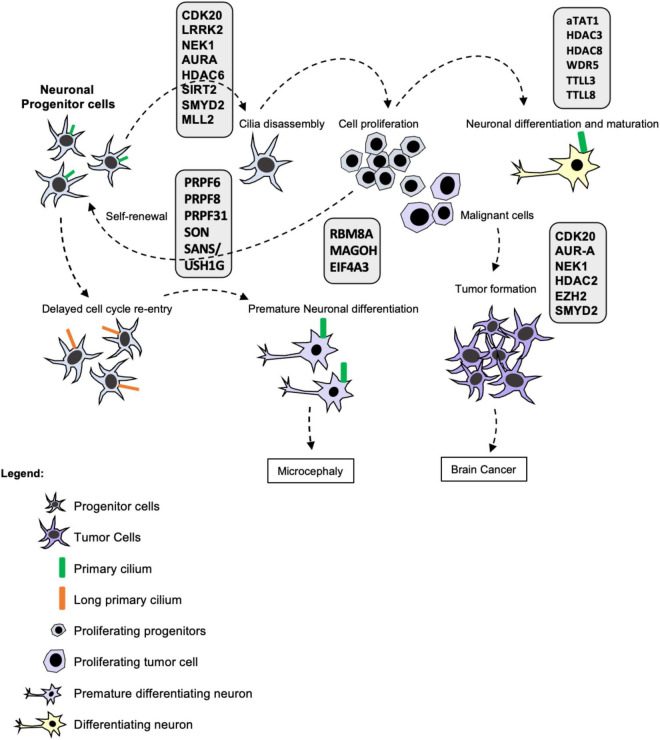
Primary cilia in brain development, maturation, and disease. Ciliogenesis is tightly coordinated with the cell cycle as primary cilia assemble and disassemble during the different phases functioning as a barrier to cell cycle progression. Whereas resting and differentiated cells form a cilium, proliferating cells such as stem cells and progenitor cells disassemble their primary cilia prior to cell division. Stem cell proliferation is important for stem cell self-renewal, maintenance of the progenitor pool and subsequent lineage commitment and differentiation. Many post-transcriptional and post-translational modifications take place in the cilium modulating its formation, stability and activity as depicted in the figure. Therefore, cilia defects and absence affect proliferation and differentiation of neural progenitors leading to developmental diseases and cancer such as Glioblastoma and Medulloblastoma. Cilia disassembly defects cause elongation delaying cell cycle re-entry leading to premature differentiation of NPCs and reducing the pool of progenitors and ultimately leading to microcephaly. Moreover, the ciliary status of neuronal cells from patients with neurodevelopmental and neurodegenerative diseases is yet to be determined. Thus, cilia function is essential for stem cell maintenance, neural development and neural function in health and disease.

Gabriel et al. (2016) demonstrated that a centrosomal protein called CPAP (centrosomal-P4.1-associated protein) plays a crucial role in cilia disassembly and maintenance of the NPC pool. This work described that CPAP provides a scaffold for the cilia disassembly complex acting as a negative regulator of ciliary length. CPAP is mutated in Seckel syndrome and is correlated with microcephaly. Seckel patient-derived fibroblasts presented long cilia associated with delayed cilia disassembly and cell cycle re-entry (Gabriel et al., 2016). iPSC-derived neuronal progenitor cells (NPCs) from Seckel patients presented signs of early differentiation such as the neuronal marker TUJ1, contained long cilia, reduced proliferative capacity and delayed G1-S transition measured by incorporation of EdU and cyclin A levels (Gabriel et al., 2016). The delay in cell cycle re-entry causes premature differentiation of NPCs reducing the pool of progenitors and ultimately leading to microcephaly (Alcantara and O’Driscoll, 2014; Gabriel et al., 2016). Thus, primary cilia are important to regulate NPC maintenance by controlling the differentiation of neuronal progenitors. Moreover, the primary cilium blocks cell cycle progression inhibiting self-renewal and allowing lineage commitment via neuronal differentiation (Figure 1).

## Cilia During Lineage Commitment and Differentiation

Primary cilia regulate the patterning and morphogenesis of the forebrain by modulating cellular division and acting as a signaling hub concentrating receptors of morphogens and downstream effectors that guide neuronal differentiation. Mouse studies have shown that mutations in genes involved in IFT that are essential for ciliogenesis and cilia maintenance like *Ift72* and *Kif3a* (Spassky et al., 2008; Gorivodsky et al., 2009) disrupted cell migration and proper formation of neuronal tissue resulting in brain patterning defects. Moreover, these alterations were associated with disrupted Hedgehog signaling showing the importance of primary cilia in modulating SHH-mediated morphogenesis. Similarly, IFT88 alterations cause severe telencephalic architectural disorganization. *Ift88* mutations were associated with cranio-facial developmental defects in mice, however, linked to impaired Wnt signaling (Tian et al., 2017).

## Ciliopathies and Neurodevelopmental Diseases

Primary cilia play important roles during brain development controlling patterning and morphogenesis of the forebrain. Abnormal cilia formation or function resulting from genetic mutations are called Ciliopathies. Ciliopathies are heterogeneous diseases that can affect almost all organs as primary cilia can be formed in every cell of the human body from stem cells (Yanardag and Pugacheva, 2021), to neurons (Dahl, 1963; Handel et al., 1999; Bishop et al., 2007), epithelial cells in different organs (Jain et al., 2010; Rocha et al., 2014), and blood and bone marrow cells (Singh et al., 2016). Thus, alterations in primary cilia can lead to a range of different phenotypes. Brain alterations observed in ciliopathies include brain malformation, microcephaly, hydrocephalies, corpus callosum agenesis, retinal degeneration, intellectual disability, and autism spectrum disorder as well as brain tumors (Table 1).

Ciliopathies present brain alterations and axonal tract defects are a common feature of human ciliopathies. Joubert Syndrome is a well-studied ciliopathy with known associated genes and characteristic brain malformations such as axonal tract defects. Guo et al. (2019) studied the effect of *Arl13b* deletion in neurons and observed axonal growth defects *in vitro.* They demonstrated that convergent signaling pathways such as PI3K, AKT and AC3 originated in the primary cilia are propagated across the neuronal cell body to modulate axonal development (Guo et al., 2019). Interestingly, this work demonstrated that ciliary receptor activation modulates growth cone dynamics and axonal development indicating that primary cilia activity is important for neuronal development and connectivity (Guo et al., 2019).

## Primary Cilia in Neurodegenerative Diseases

Parkinson’s disease (PD) is one of the most common neurodegenerative diseases characterized by degeneration of dopamine neurons from the substantia nigra and accumulation of misfolded protein aggregates (Lewy bodies) in the brain (Halliday et al., 2014). LRRK2 (leucine-rich repeat kinase 2) autosomal dominant mutations are a major risk factor for PD (Alessi and Sammler, 2018). LRRK2 is a kinase that phosphorylates a subset of Rab GTPases such as Rab8 and Rab10 that are involved in ciliogenesis (Jeong and Lee, 2020). However, Rab8A and Rab10 have opposing roles in normal ciliogenesis. Rab8A promotes cilia elongation, whereas Rab10 suppresses ciliogenesis as it binds to RILPL1 (Rab interacting lysosomal protein-like 1) protein and hinders cilia formation (Dhekne et al., 2018).

The association of LRRK2 mutation and ciliogenesis disruption was observed in PD patient-derived iPSCs that fail to form a cilium in culture (Dhekne et al., 2018). Moreover, neurons in the striatum of LRRK2 mutant mice displayed reduced number of ciliated cells and expressed lower levels of Gli1 transcripts (Dhekne et al., 2018). It is possible then that these alterations reduce neuronal response to SHH in PD patients carrying LRRK2 mutations. Since the primary cilium plays an important role in the SHH signaling pathway (Goetz and Anderson, 2010; Ho and Stearns, 2021), and SHH signals are required to generate human iPSC-derived midbrain dopaminergic neurons (Cooper et al., 2010), cilia loss due to LRRK2 mutations affects the dopaminergic neuron population.

The importance of LRRK2 in primary cilia formation was further tested in mouse embryonic fibroblast (MEF) cells expressing the pathogenic R1441C LRRK2 mutation. MEFs expressing R1441C LRRK2 at endogenous levels failed to produce a primary cilium, whereas cells treated with LRRK2 inhibitor MLi-2 were able to form cilia (Sobu et al., 2021).

Thus, accumulating evidence has uncovered a functional role for LRRK2 in primary cilia formation in murine and human models. These data highlight a molecular pathway involving cilia and SHH signals for proper neuronal function that is altered in Parkinson’s patients. Strikingly, these data show that primary cilia are important neuronal sensors not only during developmental stages but throughout differentiation and later stages of neuronal maturation and ultimately play an important role in neurodegenerative processes.

## Primary Cilia and Brain Tumors

Multiple recent studies have reported defects associated with primary cilia in various cancers (Higgins et al., 2019; Sarkisian and Semple-Rowland, 2019). As primary cilia are important for signaling and cell cycle and are assembled from centrioles that organize spindle poles, it is likely that the absence of the organelle may promote tumorigenesis by aberrant signal transduction and cell cycle regulation (Figure 1). Primary cilia are diminished or lost in multiple cancers, including pancreatic ductal adenocarcinoma (PDAC), renal cell carcinoma, basal cell carcinoma, breast cancer, ovarian cancer, prostate cancer, medulloblastoma, cholangiocarcinoma, melanoma and glioblastoma (reviewed by Higgins et al., 2019; Sarkisian and Semple-Rowland, 2019). Several studies have reported aberrant ciliogenesis in glioblastoma (GBM) lines and primary samples reviewed by Alvarez-Satta and Matheu (2018) and Sarkisian and Semple-Rowland (2019). Interestingly, primary cilia formation was reported to be disrupted at early GBM stages so that fully formed cilia were either completely absent, extremely rare or abnormal after incomplete ciliogenesis (Moser et al., 2009, 2014) indicating that primary cilia are involved in brain tumor pathogenesis.

Are cilia functionally important in glioblastomagenesis? Functional studies revealed that primary cilia loss mediated by cyclin-dependent kinase 20 (CDK20, formerly named as cell-cycle related kinase or CCRK) overexpression promotes GBM proliferation (Yang et al., 2013). CCRK behaves as an oncogene in GBM as depleting CDK20/CCRK inhibited tumor growth (Ng et al., 2007; Yang et al., 2013). Its downregulation also restored ciliogenesis thus indicating that a decrease in cilia promotes tumor growth (Yang et al., 2013). In support of this, Loskutov et al. (2018) also showed that ciliary loss is necessary to maintain a highly proliferative phenotype in GBM. This is not surprising as ciliogenesis is tightly regulated and coordinated with the cell cycle.

Further support for the tumor-suppressing role of cilia in GBM was recently reported by Goranci-Buzhala et al. (2021) who showed that patient-derived glioblastoma stem cells displayed high levels of proteins that are involved in cilia disassembly such as CPAP, Aurora-A, NEK2, NDE1 and OFD1 that resulted in suppressing ciliogenesis. Furthermore, patient-derived glioblastoma stem cells and clinical glioblastoma tissue displayed reduced primary cilia formation associated with increased cell proliferation. Importantly, rescuing ciliogenesis by depleting the disassembly protein complex restored cilia formation and non-malignant phenotype, as glioma stem cells were able to trigger differentiation (Goranci-Buzhala et al., 2021). Strikingly, ciliogenesis-induced differentiation was critical to prevent infiltration of glioma stem cell organoids into the brain of mice showing that signals from the primary cilia determine glioma stem cell fate (Goranci-Buzhala et al., 2021). Collectively, these studies highlight the importance of primary cilia in regulating neuronal differentiation, cell proliferation and, consequently, GBM tumorigenesis as cilia formation is impaired in GBM (Sarkisian and Semple-Rowland, 2019).

## Cilia and Medulloblastoma

Medulloblastoma, a tumor arising in the cerebellum, is the most common type of malignant pediatric brain tumor. Medulloblastoma is causally linked to mutations in the SHH pathway that signals through the primary cilium (Rohatgi et al., 2007; Corbit et al., 2008). *PTCH1* mutations are the most common MB driver (Northcott et al., 2017). Amplification of the Sonic Hedgehog pathway is observed in the best characterized medulloblastoma subgroup (SHH), with 30% of human tumors having mutations in Patched, Sufu (Suppressor of Fused Homolog), Smoothened, or other genes in this pathway (Northcott et al., 2011, 2017). These mutations lead to constitutively activated SHH signaling, driving tumor development and progression. SHH signaling requires the primary cilium, and both are essential for cerebellar development (Chizhikov et al., 2007; Spassky et al., 2008). SHH is critical for the ontogenesis and patterning of the cerebellum by regulating the expansion and proliferation of neural progenitor cells (Dahmane and Ruiz i Altaba, 1999; Wallace, 1999; Wechsler-Reya and Scott, 1999). Notably, cilia are only found in SHH and WNT subgroups but not present in other medulloblastoma subgroups (Han et al., 2009) thus underscoring the functional importance of cilia in tumors that are driven by SHH and WNT signaling. Furthermore, it is worth noting that the SHH and WNT subgroups have better prognosis and outcomes than the other two Medulloblastoma subgroups (Northcott et al., 2011) suggesting that the presence of primary cilia in these tumors results in a less malignant phenotype.

## Alternative Splicing of Genes Encoding Cilia Proteins

Alternative splicing affects most human multi-exon genes. As such, alternative splicing has been reported in several genes involved in ciliogenesis. Notably, a recent study looking for splicing changes in GBM subgroups identified enrichment of alternative splicing events in ciliogenesis genes when comparing mesenchymal vs. proneural GBM clusters (Guardia et al., 2020). Expression of some of these genes correlated with prognosis and survival thus underscoring their importance for GBM tumor progression and aggressiveness (Guardia et al., 2020). In our recent study of PRMT5 inhibition in patient-derived GBM stem cells, we identified transcriptome-wide altered splicing (Sachamitr et al., 2021). Notably, we observed enrichment of altered splicing in genes involved in cilia and centrosomes. CDK20 (aka CCRK) splicing was affected and found to shift to the less proliferative isoform (Sachamitr et al., 2021). In addition, we identified splice shifts in the ULK3 and STK36 kinases (listed in Table 1). ULK3 regulates SHH signaling by phosphorylating the Gli transcription factors (Maloverjan et al., 2010). Similarly, STK36 is a fused kinase homolog which also regulates SHH signaling by regulating the Gli transcription factors (Wilson et al., 2009). We also found splicing shifts in NEK1 kinase which is mutated in polycystic kidney disease and is required for ciliogenesis (Shalom et al., 2008; White and Quarmby, 2008). These results suggest that some of the anti-proliferative effects of PRMT5 inhibitors may be mediated by its impact on missplicing of ciliary kinases.

RNA splicing has also been functionally implicated in Medulloblastoma. Dubuc et al. (2012) identified alternative splicing signatures that can accurately discriminate the four different medulloblastoma subgroups with alternative splicing in SHH and group 3 being the most prevalent. The importance of splicing in medulloblastoma development is further underscored by the recent discovery of recurrent hotspot mutations in the non-coding U1snRNA found exclusively in the SHH medulloblastoma subgroup (Suzuki et al., 2019). The U1snRNA mutations inactivate tumor suppressors such as Patched and activate oncogenes like Gli genes thus functionally affecting SHH and ciliary signaling (Suzuki et al., 2019).

## Spliceosome Involvement in Ciliary Diseases Like Retinitis Pigmentosa

Heterozygous mutations in the spliceosomal genes PRPF6, PRPF8 and PRPF31 cause retinitis pigmentosa (RP, Tanackovic et al., 2011a; Table 1). Intriguingly, a recent genome-wide RNAi screen revealed these same pre-mRNA splicing factors PRPF6, PRPF8, and PRPF31 to be essential for ciliogenesis (Wheway et al., 2015). Using patient-derived cells from RP patients bearing these mutations, two independent studies revealed generalized spliceosomal defects affecting alternative splicing profiles in these cells (Tanackovic et al., 2011b; Buskin et al., 2018). PRPF31 mutations disrupt splicing of genes encoding splicing factors and ciliary proteins specifically in the retina tissues correlating with the RP phenotypes observed (Buskin et al., 2018). Furthermore, retina photoreceptor cells display unique splicing patterns, which are not found in transcripts from other cell types and are controlled by the Musashi 1 (MSI1) protein (Wheway et al., 2019). This indicates that tissue specific alternative splicing contributes to the tissue-specific ciliopathy phenotypes. Therefore, ciliopathies can emerge either by genetic variants in spliceosomal proteins, or because of variants affecting splicing of specific cilia genes (Wheway et al., 2019).

Three recent papers further cement the link between splicing factors and ciliogenesis. The splicing factor SON was reported to be required for centrosome assembly and ciliogenesis (Stemm-Wolf et al., 2021). The ciliopathy protein SANS/USH1G, mutations of which lead to Usher syndrome, was shown to regulate pre mRNA splicing and interact with splicing factors SON and SF3B1 (Yildirim et al., 2021). SANS is required for the transfer of tri-snRNPs between Cajal bodies and nuclear speckles which are critical for spliceosome assembly. Furthermore, its depletion or pathogenic SANS mutations were shown to affect the splicing of genes important for cell proliferation and Usher syndrome (Yildirim et al., 2021). An additional layer of complexity was recently discovered by Herve LeHir and colleagues who reported that the exon-junction complex (EJC) is required for ciliogenesis in neural stem cells by accumulating to ciliary bodies and recruiting mRNAs important for centrosome organization (Kwon et al., 2021). Mutations or haploinsufficiency of core EJC components Magoh, RBM8A and EIF4A3 result in microcephaly and neurogenesis defects (Silver et al., 2010; Mao et al., 2015, 2016). All this underscores the functional importance of core splicing factors and RNA localization and processing components in ciliopathies and neurogenesis.

## Posttranslational Modification Enzymes With a Role in Ciliogenesis Are Linked to Disease

Cilia has a very unique structure as they are formed by nine peripheral pairs of microtubules that are subjected to the activity of posttranslational modifications (PTMs) enzymes (Ishikawa and Marshall, 2011; Janke and Bulinski, 2011). Tubulin undergoes several highly conserved PTMs including acetylation, detyrosination, glutamylation, and glycylation (Janke and Bulinski, 2011). These PTMs accumulate on a subset of microtubules that are long-lived, including those of cilia and centrioles. Interestingly alterations of PTMs levels have been linked to ciliogenesis defects and tumor formation in mouse models and human samples showed a correlation to tumor progression in murine model for colorectal cancer and samples from patients (Rocha et al., 2014).

Alterations in the level of axonemal glutamylation affect *SHH* signaling as it interferes with the translocation of GLi3 and tethering of Polycystic Kidney Disease 1/2 (PKD1/2) (He et al., 2018) and impairs the entry of Smoothened and Gli3 into the cilia (Hong et al., 2018). Additionally, glutamylation was also associated with cancer. A study screened for genes with correlation for brain metastasis by microarray and showed that TTLL4, a tubulin glutamylase enzyme, overexpression in breast cancer cells is associated with brain metastasis (Arnold et al., 2020).

Tubulin glycylation is specific to cilia (Bre et al., 1994) and glycylation enzymes are required for primary (TTLL3) and motile cilia (TTLL3 and TTLL8) formation, stability and maintenance (Xia et al., 2000; Wloga et al., 2009, 2010; Pathak et al., 2011; Bosch Grau et al., 2013; Rocha et al., 2014). Gadadhar et al. (2017) showed that glycylation accumulates in primary cilia in a length-dependent manner, and alterations of glycylating enzymes TTLL3 and TTLL8 modulates the length of primary cilia in cultured cells (Gadadhar et al., 2021).

Alterations of glycylation are linked to ciliopathies and cancer. Lack of TTLL3 reduces the number of ciliated cells and increases cell proliferation correlating to tumor progression in a mouse model for colorectal cancer (CRC) showing the importance of glycylation in cilia maintenance (Rocha et al., 2014). Analysis of several CRC cell lines also showed decrease of TTLL3 expression (Campbell et al., 2002; Ikegami et al., 2007) and of primary cilia formation (Rocha et al., 2014). Loss of glycylation was also linked to alterations in all ciliated tissues developing the hallmarks of ciliopathies such as infertility (Campbell et al., 2002; Ikegami et al., 2007; Lee et al., 2013; Konno et al., 2016; Gadadhar et al., 2021) and alterations in respiratory tract (Ikegami et al., 2010), retina (Bosch Grau et al., 2017), and brain ventricles (Bosch Grau et al., 2013).

Acetyl-K40, one of the most conserved tubulin PTMs, is the most abundant tubulin PTM in cilia (Akella et al., 2010). Acetyl-K40 marks long-lived microtubules, including those in the axonemes and basal bodies (Piperno and Fuller, 1985). Tubulin acetylation is important for regulating microtubule architecture and maintaining microtubule integrity. The acetyltransferase responsible for this modification is aTAT1. Abnormal levels of this modification are linked to neurological disorders, cancer, heart diseases and other pathological conditions, thereby yielding important therapeutic implications (Li and Yang, 2015).

## Histone Deacetylases

HDAC6 is a histone deacetylase that deacetylates the Acetyl-K40 mark in α-tubulin and regulates microtubule-dependent cell motility (Hubbert et al., 2002). Accumulating evidence shows HDAC6 is required for cilium disassembly (Pugacheva et al., 2007). Knockdown of HDAC6 increases the frequency of primary cilia or make them longer, whereas overexpression lead to fewer or shorter cilia (Pugacheva et al., 2007; Yang et al., 2014; Bangs et al., 2015; Ran et al., 2015). HDAC6 also deacetylates cortactin, and this activity also shortens the cilium, by promoting actin polymerization around the base of the primary cilium (Ran et al., 2015). In addition, HDAC6 is involved in autophagy, clearance of misfolded proteins and tau-mediated neurodegeneration (Yu et al., 2016). SIRT2, an NAD-dependent deacetylase, can also deacetylate a-tubulin (North et al., 2003). Furthermore, SIRT2 regulates ciliogenesis and centrosome amplification (Zhou et al., 2014). Overexpression of SIRT2 in renal epithelial cells disrupted cilia formation, causing decreased numbers of cells with cilia and decreased cilia length, while inhibition of SIRT2 activity blocked cilia disassembly during the cell cycle (Zhou et al., 2014). HDAC2 is essential for suppression of cilia formation by positively regulating Aurora A levels in dividing tumor cells (Kobayashi et al., 2017). In contrast, HDAC3 and HDAC8 are required for cilium assembly and elongation (Park et al., 2019; see Table 1).

## Methyltransferases

Other PTM enzymes involved in ciliogenesis are the methyltransferases EZH2 and WDR5. Zingg et al. (2018) showed that EZH2 is a melanoma driver by deconstructing the primary cilia through silencing of cilia genes. They showed that gain of EZH2 promotes loss of primary cilia in benign melanocytic lesions (Zingg et al., 2018). In contrast, blockade of EZH2 activity evokes ciliogenesis and cilia-dependent growth inhibition in malignant melanoma (Zingg et al., 2018). Subsequently, loss of cilia enhances pro-tumorigenic WNT/β-catenin signaling, which drives metastatic melanoma in benign cells.

WDR5 regulates left-right patterning and controls ciliogenesis by acting as a scaffold rather than its histone methyltransferase activity (Kulkarni and Khokha, 2018). In addition, WDR5 stabilizes apical actin architecture to promote multiciliated cell formation (Kulkarni et al., 2018). Specifically, WDR5 binds to basal bodies and migrates apically, where F-actin organizes around WDR5. This function was found to be independent to its chromatin modification activity (Kulkarni et al., 2018). Furthermore, the H3K4 methyltransferase MLL2, which is part of the WDR5 complex, suppresses ciliogenesis through regulating actin dynamics and vesicle transport (Yang et al., 2019). Another methyltransferase, SMYD2, was also recently identified as an α-tubulin methyltransferase involved in ciliogenesis (Li et al., 2020). Depletion of SMYD2 led to increased cilia assembly and elongation (Li et al., 2020). In addition, inhibition of the G9A/GLP H3K9 methyltransferase with inhibitor BIX-01294 was found to induce autophagy and promote cilia elongation in human retina pigmented epithelial cells (Shin et al., 2015). Mutations in several methyltransferase enzymes have neuronal phenotypes such as Weaver syndrome for EZH2 (Gibson et al., 2012), Kabuki syndrome for MLL2 (Ng et al., 2010) and Kleefstra syndrome for the EHMT1/GLP1 gene (Kleefstra et al., 2006) underscoring their importance in neuronal development (Table 1).

## Future Directions in Cilia Studies Using Induced Pluripotent Stem Cells as Tools for Drug Discovery

Given the role of primary cilia in neuronal differentiation and tumorigenesis, studies investigating the mechanisms underlying ciliogenesis and its impact on neuronal biology will tremendously benefit the development of therapeutic strategies for the treatment of ciliopathies and several neuronal pathologies including both neurodevelopmental and neurodegenerative diseases, as well as brain cancers. induced pluripotent stem cells (iPSCs) offer a unique platform to study human primary cilia formation and activity during neuronal differentiation, maturation and aging as primary cilia play important roles on neuronal tissue patterning, maturation, survival and tumorigenesis by orchestrating and coordinating important developmental signaling pathways such as WNT and SHH (Youn and Han, 2018).

The advantages of using human iPSCs include: (1) to obtain human neuronal cells that are otherwise rare samples and difficult to obtain for research, (2) the opportunity to study developmental processes such as differentiation and maturation *in vitro* using appropriate culture media, and (3) the possibility to obtain cells from patients allowing the investigation of cellular mechanisms in pathological contexts. Disease-related mutations can also be introduced by CRISPR-Cas9 technology mimicking various patient genetic backgrounds. In this context, large compound libraries can be tested on iPSC-derived cells allowing the screening for compounds that have an impact on ciliogenesis, maintenance and activity. Finally, (4) iPSCs can be used to generate tissue-specific 3D cultures called organoids to model human tissue development and disease. Organoids provide the opportunity to investigate architectural arrangement, cellular signaling events and molecular mechanisms involved in neurodevelopmental and neurodegenerative disorders, including Ciliopathies, using rare patient-derived samples.

Brain organoids can be obtained by cultivating pluripotent stem cells in 3D suspension and are being used to model a great variety of organs including gut, retina, and brain (Hofer and Lutolf, 2021). The generation of brain organoids first described by Lancaster et al. (2013) opened an avenue for the development and modeling of brain-specific tissue allowing the study not only of developmental disorders such as ASDs (Maussion et al., 2019), but also degenerative processes such as PD (Mohamed et al., 2021) and Retinitis Pigmentosa (Lane et al., 2020). Importantly, as described in this review, primary cilia play critical roles during brain development and tumorigenesis. Using brain organoids to study how genetic and epigenetic alterations modulate these processes using patient-derived cells with disease-specific mutations will allow for unprecedented advances in personalized medicine and drug screening for neurological disorders.

## Conclusion

The primary cilium plays crucial roles in modulating cellular signaling pathways that are essential for stem cell maintenance and neural development. Interestingly, ciliary defects are observed in developmental and neurodegenerative diseases, and brain cancers such as Glioblastoma and Medulloblastoma. Moreover, neuronal phenotypes such as microcephaly and intellectual disability are a common feature of human ciliopathies. Ciliogenesis is controlled by cellular processes such as the cell cycle, epigenetics factors, post-translational modifications as well as RNA splicing. This regulation has a strong impact on neuronal development as evidenced by the plethora of neuronal phenotypes observed in enzymatic deficiencies and mutations of epigenetics and PTM enzymes as well as RNA processing components. This role seems to be equally important as the more traditional roles of these enzymes on chromatin dynamics, gene expression and posttranscriptional control.

Moreover, a growing body of evidence is unraveling the importance of the primary cilium in neuronal development, activity and maintenance as demonstrated by human iPSCs-based studies strongly implicating cilia in neurodevelopment, neurodegenerative diseases, and brain cancers. This emphasizes the importance of iPSCs studies in understanding human neuronal pathologies as they provide a platform for modeling human neuronal diseases. Thus, understanding the epigenetic and PTM mechanisms of how cilia are formed, maintained and disassembled is fundamental for neuroscience research and drug discovery.

## Author Contributions

CR and PP contributed to conception, design, and writing of the review. Both authors contributed to manuscript revision, read, and approved the submitted version.

## Conflict of Interest

The authors declare that the research was conducted in the absence of any commercial or financial relationships that could be construed as a potential conflict of interest.

## Publisher’s Note

All claims expressed in this article are solely those of the authors and do not necessarily represent those of their affiliated organizations, or those of the publisher, the editors and the reviewers. Any product that may be evaluated in this article, or claim that may be made by its manufacturer, is not guaranteed or endorsed by the publisher.
